# Caring for students with disabilities: a SEM analysis of compassion fatigue, emotional regulation, and professional resilience among Saudi special education teachers

**DOI:** 10.3389/fpsyg.2026.1686149

**Published:** 2026-02-16

**Authors:** Houcine Benlaria, Adel Saber Alanazi

**Affiliations:** 1 College of Business, Jouf University, Sakakah, Saudi Arabia; 2 King Salman Center for Disability Research, Riyadh, Saudi Arabia; 3 College of Education, Jouf University, Sakakah, Saudi Arabia

**Keywords:** compassion fatigue, emotional regulation, inclusive education, professional resilience, Saudi Arabia, special education teachers, students with disabilities, teacher well-being

## Abstract

**Introduction:**

This study investigated the relationships between compassion fatigue, emotional regulation, and professional resilience in Saudi special education teachers, examining how these factors influence the quality of care for students with disabilities within the context of Saudi Arabia’s ongoing inclusive education reforms under Vision 2030.

**Methods:**

Drawing on a nationally diverse sample of 412 special education teachers from six regions of the Kingdom, we examined how work demands and personal resources shape key professional outcomes through psychological mechanisms relevant to teacher well-being and instructional effectiveness within a rapidly reforming institutional and policy environment. A structural modeling approach was used to analyze validated measures of work demands, personal resources, compassion fatigue, emotional regulation, professional resilience, professional efficacy, and job satisfaction.

**Results:**

Work demands were positively associated with compassion fatigue (*β* = 0.521, *p* < 0.001), whereas personal resources were positively associated with emotional regulation (*β* = 0.210, *p* < 0.01). Compassion fatigue was negatively related to professional resilience (*β* = −0.295, *p* < 0.001), while emotional regulation was positively related to resilience (*β* = 0.394, *p* < 0.001). Professional resilience strongly predicted professional efficacy (*β* = 0.610, *p* < 0.001) but did not significantly predict job satisfaction. Compassion fatigue partially mediated the relationship between work demands and resilience, accounting for 45.3% of the variance, while emotional regulation partially mediated the relationship between personal resources and resilience, accounting for 37.6% of the variance. Sequential mediation pathways indicated that professional efficacy is shaped primarily through teachers’ psychological resilience rather than through general job satisfaction.

**Discussion:**

These findings suggest that supporting teachers’ psychological well-being is fundamental to ensuring high-quality care for students with disabilities and to the sustainability of inclusive education initiatives in Saudi Arabia. From a policy and practice perspective, the results highlight the importance of integrating emotional regulation training, workload management, and resilience-building programs into teacher professional development frameworks to support the successful implementation of inclusive education reforms within the Saudi educational system. Future research should use longitudinal and experimental designs to study these relationships’ causal dynamics. Comparative multi-context studies are also recommended to evaluate the model’s generalizability across educational systems and institutional policy settings.

## 1 Introduction

Special education practitioners in Saudi Arabia operate within a multifaceted professional environment characterized by significant emotional demands and rapidly evolving educational standards, especially as the Kingdom advances its extensive inclusive education initiatives under Vision 2030 (Albash, 2024; Alsolami, 2024; Alshahrani and Abu-Alghayth, 2025). Recent policy analyses indicate that these reforms have expanded teachers’ instructional, emotional, and administrative responsibilities, often without commensurate growth in psychological support systems or workload mitigation strategies (Alrudayni, 2025; Alharbi and Iqtadar, 2025). Educators supporting students with disabilities—such as those with autism spectrum disorder (ASD), intellectual disabilities, or multiple impairments—face the dual challenge of meeting diverse academic needs while navigating complex emotional dynamics and systemic limitations inherent in modern special education practices. In inclusive school settings, teachers are increasingly expected to individualize instruction, manage behavioral complexities, collaborate closely with families, and comply with evolving accountability frameworks, all of which intensify daily work pressures. This demanding professional context increases the susceptibility of special education teachers to elevated occupational stress, substantially heightening their risk of severe psychological outcomes, including burnout and compassion fatigue (Antoniou et al., 2024; Amarneh and Al-Dwieb, 2022).

Compassion fatigue, a complex phenomenon involving emotional exhaustion and secondary traumatic stress, represents a significant challenge in special education, as it undermines educator well-being and professional efficacy, thereby reducing the quality of educational services for students with disabilities (Purnamasari et al., 2020; Shubair et al., 2023). Within the Saudi context, inclusive education reforms have placed special education teachers at the intersection of heightened emotional labor and systemic transition, increasing vulnerability to compassion fatigue when institutional resources and organizational support structures are insufficient (Alharbi and Iqtadar, 2025). The emotional labor inherent in special education is intensified by increasing student caseloads, diminishing institutional resources, and the ongoing need to collaborate with families facing their own psychological challenges (Dotimas, 2022; Oyan et al., 2024). For teachers working with students who require sustained emotional attunement and behavioral support, prolonged exposure to these stressors may erode empathy, reduce emotional availability, and compromise the quality of care provided. The convergence of these factors underscores the need for an in-depth examination of both the risk factors contributing to educator distress and the psychological mechanisms that may mitigate the adverse effects of chronic work-related stress.

Personal resources, such as self-compassion, emotional regulation skills, and professional self-efficacy, are crucial for enhancing educators’ professional resilience and promoting sustained job satisfaction and career longevity (Golparvar and Parsakia, 2023; Parveen and Pal, 2024). Emotional regulation is an essential cognitive skill that enables educators to effectively manage work-related stressors without succumbing to severe emotional exhaustion or professional burnout (Hu, 2023; Abdel Hadi and Gharaibeh, 2023). In special education classrooms, effective emotional regulation allows teachers to respond adaptively to challenging student behaviors, maintain instructional consistency, and preserve a supportive learning climate. However, the development and practical application of such psychological resources do not occur in a social vacuum; instead, institutional norms, organizational practices, and professional role expectations substantially shape how educators in Saudi Arabia perceive stress, interpret challenges, and respond to professional adversity (Findling et al., 2023; Işık and Seymen, 2023). Broader socio-professional values emphasizing emotional restraint, collective responsibility, and professional commitment may further influence how teachers internalize stress and seek support, highlighting the importance of context-sensitive approaches to understanding teacher well-being.

Despite the proliferation of international research examining teacher burnout and resilience across various educational contexts, the unique institutional and policy environment of special education in the Middle East—particularly in Saudi Arabia—remains substantially underexplored in the empirical literature (Oyan et al., 2024). Recent scholarship on inclusive education policy in Saudi Arabia has primarily focused on structural and curricular reform, with comparatively limited attention to teachers’ psychological well-being and emotional sustainability (Alrudayni, 2025). Existing theoretical models and intervention frameworks, which have been predominantly developed in Western contexts, often fail to adequately account for the complex interplay between contextual constraints, institutional structures, and psychological mechanisms, limiting their applicability and effectiveness in reform-driven educational systems. This significant gap in the literature underscores the need for contextually grounded empirical research to inform the development of appropriate support systems and interventions.

To address this critical research gap, the present study develops and empirically tests a contextually grounded structural model that systematically integrates four key theoretical perspectives: Compassion Fatigue Theory, Conservation of Resources (COR) Theory, Emotional Regulation Theory, and Resilience Theory. Using advanced structural equation modeling (SEM) techniques, this study examines the complex pathways through which work demands, personal resources, and emotional regulation interact to influence the development of compassion fatigue, professional resilience, and key professional outcomes, including job satisfaction and professional efficacy, among Saudi special education practitioners. By linking these psychological processes to the broader goals of inclusive education under Vision 2030, the study aims to generate evidence-based insights that can inform teacher support policies, professional development programs, and sustainable workforce strategies within the Saudi educational system.

## 2 Research objectives and significance

This research is guided by four primary objectives that collectively address significant gaps in the special education literature. First, the study aims to assess the prevalence of, and identify key predictors for, compassion fatigue among Saudi special education teachers, providing essential baseline data for this underexplored population within a rapidly transforming inclusive education system. Second, it seeks to determine the most salient personal resources that effectively promote emotional regulation and resilience in response to the expanding professional demands associated with inclusive education reforms in Saudi Arabia. Third, the research examines the mediating role of emotional regulation in the relationship between occupational stress and professional resilience, thereby contributing to a theoretical understanding of these psychological processes in non-Western educational contexts. Finally, the study investigates how institutional norms, policy-driven role expectations, and workforce structures influence the manifestation and interpretation of these psychological constructs, offering insights into contextual factors shaping educator well-being that are often overlooked in international models of teacher stress and resilience.

The practical significance of this work lies in its potential to inform the development of **i**nstitutionally responsive interventions aimed at enhancing teacher well-being, reducing burnout, and supporting sustainable professional growth in special education settings, particularly as schools adapt to inclusive education mandates under Saudi Vision 2030. Recent policy-oriented research emphasizes that the success of inclusive education reforms depends not only on structural and curricular change but also on the psychological sustainability of the teaching workforce (Alrudayni, 2025; Alharbi and Iqtadar, 2025). By examining the institutional and professional context influencing educator well-being in Saudi Arabia, this research contributes to the global effort to create more supportive and sustainable special education workforces while responding directly to national policy priorities.

### 2.1 Research contributions

This study makes several significant contributions to the special education literature and practice. Theoretically, it represents the first comprehensive empirical examination of the integrated relationships among work demands, personal resources, compassion fatigue, emotion regulation, and professional outcomes within the context of Saudi Arabia’s inclusive education reforms. By situating established psychological theories within a Saudi institutional and policy framework, the study extends their applicability beyond Western educational settings through contextual validation rather than cultural moderation. Methodologically, the study employs advanced SEM techniques to test complex mediation pathways, offering insights into the psychological mechanisms underlying educator well-being and highlighting the differential pathways through which professional efficacy and job satisfaction are shaped. Practically, the findings inform evidence-based policy development and intervention design specifically tailored to the Saudi educational context, supporting the Kingdom’s Vision 2030 inclusive education objectives through targeted recommendations for teacher training, workload management, and resilience-building initiatives.

By addressing these theoretical gaps and practical needs, this research advances the global understanding of special education practitioners’ well-being and provides contextually grounded and policy-relevant insights to inform the development of more effective, contextually appropriate support systems for educators working with students with disabilities across diverse educational systems and rapidly reforming institutional environments.

## 3 Literature review

### 3.1 Psychological mechanisms and theoretical foundations

#### 3.1.1 Compassion fatigue and professional burnout in special education

Compassion fatigue, defined as the emotional residue resulting from prolonged interactions with individuals in distress, has become a significant issue for special education professionals (Jeanmonod et al., 2024). This multidimensional construct encompasses burnout—when chronic workplace stress leaves individuals feeling emotionally drained and cynical—and secondary traumatic stress—when exposure to students’ prolonged distress induces trauma-like symptoms (Mathias and Wentzel, 2017; Tang et al., 2023). Recent cross-cultural studies illustrate the widespread occurrence of compassion fatigue within helping professions. Bozdag et al. (2025) reported substantial levels among neonatal nurses in palliative care settings, while Gonçalves and Gaudêncio (2023) observed similar trends among Portuguese healthcare professionals, highlighting its universal presence across diverse professional and cultural contexts.

In educational settings, compassion fatigue arises when educators become emotionally inundated by the intricate academic, behavioral, and emotional challenges faced by students (Oberg et al., 2023). Special education teachers are particularly vulnerable because they work with students with severe disabilities, respond to crises, and engage with families experiencing high levels of stress. Cuadra’s (2023) qualitative study highlighted the unique challenges faced by special education teachers in the “sandwich generation,” who must balance professional responsibilities with personal caregiving duties. Research consistently identifies heavy caseloads, insufficient resources, administrative pressures, and exposure to student trauma as primary risk factors (Dotimas, 2022), whereas adequate training, social support, and effective coping strategies serve as protective factors. Notably, Ragni et al. (2023) illustrated the protective role of self-compassion against burnout among Italian special education teachers, emphasizing the importance of individual psychological resources in mitigating the onset of compassion fatigue.

In the Saudi Arabian context, inclusive education reforms have intensified teachers’ exposure to emotional labor by expanding classroom diversity and accountability expectations, often without parallel increases in institutional support (Alharbi and Iqtadar, 2025; Alrudayni, 2025). This systemic imbalance may heighten the risk of compassion fatigue among special education teachers, underscoring the importance of examining compassion fatigue not only as an individual psychological outcome but also as a structural consequence of education reform.

#### 3.1.2 Conservation of resources theory and work demands

The COR theory offers a comprehensive framework for analyzing the interaction between work demands and personal resources and their impact on psychological outcomes (Bakker and Demerouti, 2017). This theory asserts that individuals inherently seek to acquire, maintain, and safeguard valued resources, and they experience psychological stress when these resources are threatened, depleted, or insufficiently replenished after investment. Bakker and Demerouti’s (2017) longitudinal study provides substantial evidence for the applicability of COR theory in educational settings, illustrating the impact of job demands and personal resources on work engagement and burnout trajectories over time. The findings indicate that when demands consistently exceed available resources, educators experience progressive resource depletion, resulting in adverse outcomes such as burnout and diminished job performance.

In special education contexts, work demands are factors that systematically undermine or deplete psychological resources. Işık and Seymen (2023) conducted a thorough analysis of the challenges faced by special education teachers, highlighting various work-related stressors, such as inadequate resources, excessive workloads, and insufficient administrative support. Dotimas (2022) reported that special education teachers experience significantly higher levels of work-related stress than their general education counterparts. In contrast, personal resources—including professional competence, social support networks, training opportunities, and individual coping skills—serve as protective assets that can be effectively utilized to manage demands and maintain psychological well-being. Research consistently shows that educators with greater personal resources demonstrate higher resilience and a lower likelihood of experiencing burnout compared to those with limited access to such resources.

From a COR perspective, recent analyses of Saudi inclusive education policy suggest that rapid systemic change may accelerate resource depletion when teacher support mechanisms are underdeveloped, thereby amplifying stress-related outcomes (Alrudayni, 2025). This reinforces the relevance of COR theory for understanding how policy-driven work demands translate into psychological strain among Saudi special education teachers.

#### 3.1.3 Emotional regulation and psychological well-being

Emotional regulation refers to the psychological processes by which individuals manage their emotional experiences, timing, and expression. It is widely recognized as a crucial factor in occupational well-being (Abdel Hadi and Gharaibeh, 2023; Chen and Yeung, 2023). Recent studies have furthered understanding of the essential role of emotional regulation in high-stress professional environments. Hu (2023), in a study on teacher self-compassion, emotional regulation, and emotional labor strategies as predictors of resilience in English as a Foreign Language contexts, found that effective emotional regulation strategies significantly predicted increased professional resilience and decreased burnout. Similarly, Palomera et al. (2022) conducted cross-cultural research during the COVID-19 pandemic, revealing significant differences in emotional regulation strategies between Polish and Spanish populations, thereby highlighting the importance of cultural context in understanding these regulatory processes.

The relationship between emotional regulation and resilience shows consistent patterns across various populations and challenging situations. Clarista and Puspitasari (2023) identified significant positive correlations between emotional regulation and resilience among sexual violence survivors in East Java. Similarly, Javed et al. (2022) found comparable associations among women diagnosed with polycystic ovarian syndrome. Their findings indicate that emotional regulation serves as a crucial mechanism for maintaining psychological well-being in diverse challenging situations. Research in education on emotional regulation has provided significant insights. Thomas and Zolkoski (2020) underscore the interrelatedness of emotional intelligence, resilience, and emotional regulation in mitigating stress among undergraduates, emphasizing the collective importance of these psychological constructs for academic and professional success.

In Saudi educational settings, cultural norms emphasizing emotional restraint and professional composure may shape how emotional regulation strategies are enacted and internalized by teachers, potentially strengthening their role as protective mechanisms against occupational stress (Burger and Arampatzi, 2025). This cultural dimension underscores the need to examine emotional regulation as both a psychological skill and a context-dependent adaptive strategy.

#### 3.1.4 Professional resilience and adaptation mechanisms

Professional resilience refers to the ability to recover from adversity, adapt to challenging situations, and maintain effectiveness in the face of significant stress (Al-Shomrani et al., 2024). Current research conceptualizes resilience as a dynamic and developable process rather than a fixed personality trait, emphasizing its potential for enhancement through targeted experiences and interventions. Studies on healthcare in Saudi Arabia provide valuable regional insights into resilience patterns. Alzahrani et al. (2022) explored the relationships between resilience and work-related stress among mental health nurses in Jeddah, identifying significant associations between resilience levels and job performance. Similarly, Balay-odao et al. (2021) examined hospital preparedness, resilience, and psychological burden among clinical nurses in Riyadh during the COVID-19 pandemic, highlighting cultural and contextual factors that influence resilience development in the Saudi context.

Research has also identified several factors that promote professional resilience, including a strong professional identity, effective coping strategies, supportive social networks, and opportunities for ongoing professional growth. Lloyd and Campion (2017) highlighted the importance of self-care and resilience in veterinary nursing, demonstrating that proactive strategies for fostering resilience can effectively reduce burnout and support long-term career sustainability. Resilience is especially critical for special education practitioners, given the chronic job-related stressors and substantial emotional demands inherent in their work. Resilient educators are better able to maintain student engagement, implement effective instructional strategies, and experience higher job satisfaction and career longevity.

Within Saudi Arabia’s inclusive education landscape, resilience may function as a key sustainability mechanism, enabling teachers to adapt to policy-driven change while maintaining instructional effectiveness (Alshahrani and Abu-Alghayth, 2025).

### 3.2 Cultural context and professional outcomes in Saudi special education

#### 3.2.1 Saudi Arabian educational and healthcare context

Research conducted within Saudi Arabian educational and healthcare systems provides essential contextual understanding for examining the psychological experiences of helping professionals in the Kingdom. Studies on compassion fatigue among physicians in the Makkah region (Alharbi et al., 2023) documented substantial levels of burnout and secondary traumatic stress, while research on nursing professionals (Alreshidi and Rayani, 2023) identified key predictors of compassion satisfaction and fatigue within the Saudi healthcare context. These healthcare environments share fundamental similarities with special education settings, including elevated emotional demands, limited resources, and the need to provide compassionate care to vulnerable populations. Amarneh and Al-Dwieb (2022) found significant relationships between compassion fatigue, compassion satisfaction, and resilience among intensive care unit nurses—findings that may be applicable to special education contexts given comparable professional demands and cultural considerations.

Research on professional quality of life among educators serving special populations has revealed important contextual patterns. Purnamasari et al. (2020) examined professional quality of life among teachers of children with special needs, documenting significant variations in compassion satisfaction, burnout, and secondary traumatic stress, highlighting the importance of understanding these constructs within specific cultural and educational frameworks. Darawsheh et al. (2023) provided comprehensive outlines of professional quality characteristics among special needs educators, highlighting the critical importance of adequate preparation, ongoing support, and professional development opportunities, while underscoring the complex interplay between individual characteristics and organizational factors in determining professional outcomes.

Recent Saudi-focused scholarship emphasizes that inclusive education reforms have progressed more rapidly than the development of teacher-centered support infrastructures, potentially exacerbating psychological strain among special education practitioners (Alharbi and Iqtadar, 2025; Alrudayni, 2025).

#### 3.2.2 Emotional regulation resources and cultural factors

Contemporary research has substantially advanced understanding of the factors influencing emotional regulation within cultural contexts. Abdel Hadi and Gharaibeh (2023) examined the role of self-awareness in predicting emotional regulation difficulties among faculty members and found significant relationships between levels of self-awareness and emotional regulation competencies. Their findings underscore the importance of self-reflection and metacognitive awareness in developing effective regulatory strategies. Similarly, Hasnayanti and Puspitasari (2023) investigated the relationship between social support and emotional regulation among healthcare personnel during the COVID-19 pandemic. They demonstrated that social support significantly predicted both emotional regulation abilities and overall resilience, highlighting the crucial role of interpersonal resources in maintaining psychological well-being during challenging circumstances.

Research on interventions aimed at enhancing emotional regulation and resilience has further provided valuable insights for professional development. Cheng et al. (2024) evaluated the positive effects of parent–child group emotional regulation and resilience training, demonstrating significant improvements in emotional regulation abilities and psychological well-being, and highlighting the effectiveness of targeted training programs in enhancing regulatory skills. Ghorbani Amir et al. (2019) examined the effectiveness of acceptance and commitment therapy on cognitive-emotional regulation, resilience, and self-control strategies, revealing significant improvements across all measured constructs and supporting the potential of evidence-based interventions to enhance emotional regulation and resilience among helping professionals.

#### 3.2.3 Professional efficacy and job satisfaction outcomes

Professional efficacy is a key outcome variable in educational research, encompassing teachers’ beliefs about their ability to teach effectively and support student learning. Research consistently shows that educators with strong efficacy beliefs engage in more effective pedagogical practices, demonstrate greater persistence in the face of challenges, and achieve superior student outcomes (Olawale and Hendricks, 2024; Rastegar and Rahimi, 2023). For special education professionals, efficacy beliefs are especially important due to the complex nature of their work and the need to maintain optimism and perseverance when supporting students who may show minimal observable progress. Studies on professional competence among special education teachers have identified several critical factors that influence efficacy beliefs, including thorough preparation, ongoing support, and favorable working conditions (Yao and Wang, 2024).

Extensive research has also examined job satisfaction among teachers. These studies indicate that multiple factors can affect teachers’ job satisfaction, including working conditions, administrative support, compensation structures, and a sense of professional accomplishment (Huang et al., 2024; Xu et al., 2025). Nonetheless, the significance of these factors varies considerably across cultural contexts, making cultural considerations vital for understanding job satisfaction patterns. Cross-cultural studies examining healthcare professionals in different countries have revealed that cultural values, organizational structures, and societal expectations profoundly affect the determinants of professional satisfaction. This underscores the imperative for culturally informed methodologies to comprehend and enhance professional outcomes.

#### 3.2.4 Integration of psychological constructs and cross-cultural considerations

Recent research has increasingly emphasized the interconnectedness of psychological constructs, with mediation analyses providing important insights into the mechanisms underlying educator well-being. Studies demonstrate that resilience, emotional regulation, and self-efficacy function as interrelated processes that jointly shape psychological outcomes (Joharian et al., 2024; Fitri et al., 2025). These findings support the use of integrative structural models to examine teacher well-being rather than isolated variable approaches.

Cross-cultural research further indicates that emotional regulation and resilience are shaped by cultural values, social norms, and religious beliefs. In non-Western contexts, including Saudi Arabia, collectivism, family orientation, and expectations of emotional restraint may influence how educators experience and manage occupational stress. Within the framework of Saudi Arabia’s Vision 2030 education reforms, these cultural factors interact with heightened institutional demands, reinforcing the need for culturally grounded models that capture the mediating roles of emotional regulation and resilience in special education settings (Alrudayni, 2025; Alharbi and Iqtadar, 2025).

### 3.3 Research framework

This study integrates four complementary theories to examine the well-being of special education practitioners. Compassion Fatigue Theory explains how continuous exposure to student suffering can lead to emotional exhaustion and secondary traumatic stress (Amarneh and Al-Dwieb, 2022; Figley, 1995; Oberg et al., 2023). The COR Theory posits that stress arises when psychological resources are threatened or depleted, whereas resource investment can promote well-being (Golparvar and Parsakia, 2023; Lloyd and Campion, 2017). Emotional Regulation Theory describes how effective emotion management strategies buffer work-related stress and support adaptive coping (Gross and John, 2003; Hu, 2023). Resilience Theory conceptualizes resilience as a dynamic adaptation process, enabling individuals to withstand adversity while maintaining professional functioning (Balay-odao et al., 2021; Cheng et al., 2024; Chen and Yeung, 2023; Oyan et al., 2024).

**Figure 1:**
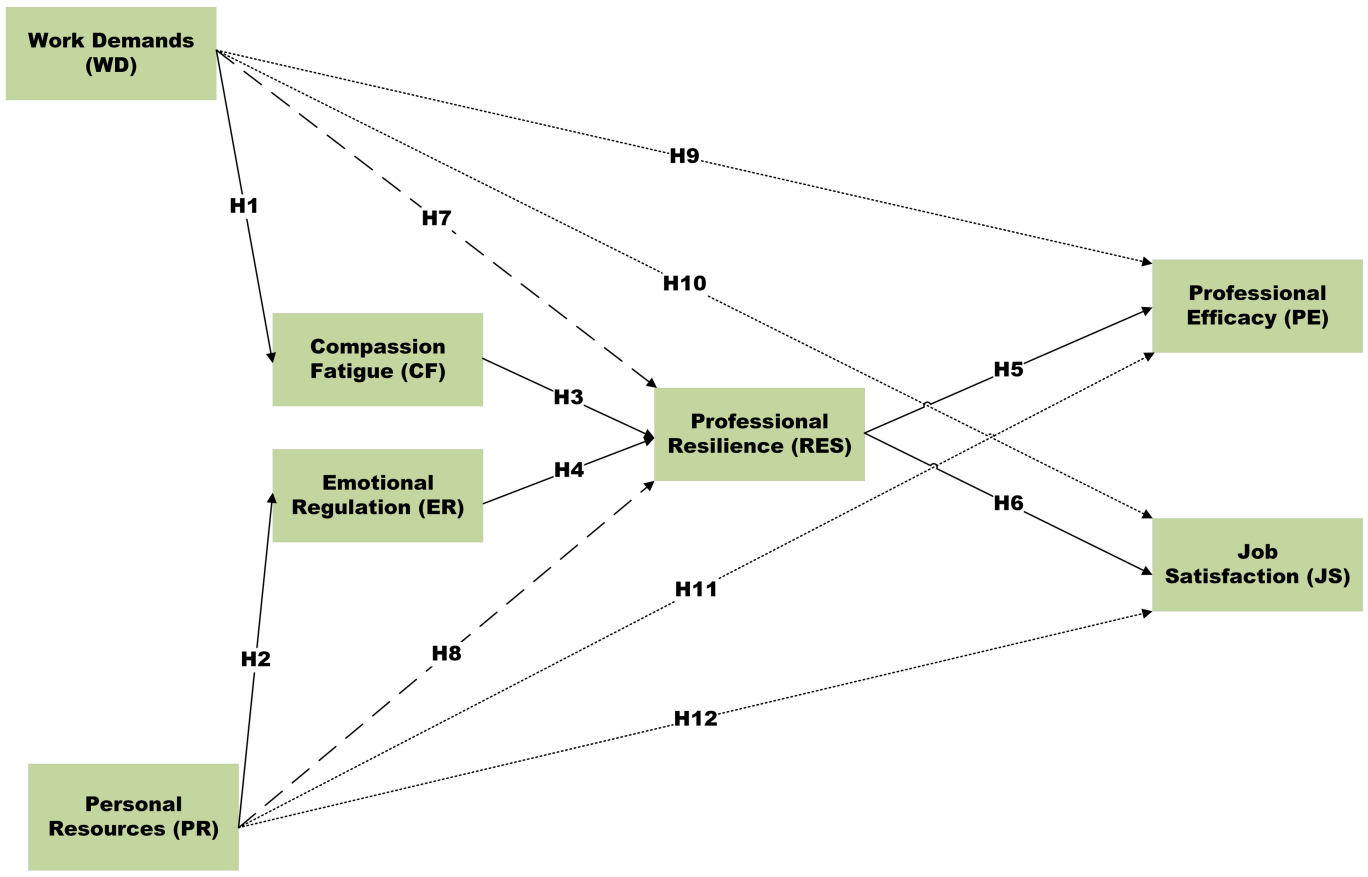
Research framework.

As illustrated in Figure 1, the theoretical model positions work demands and personal resources as exogenous variables that influence professional outcomes—operationalized in this study as professional efficacy and job satisfaction—through psychological mediators. Work demands are hypothesized to increase compassion fatigue, thereby reducing professional resilience, whereas personal resources are expected to enhance emotional regulation, thereby increasing resilience. Professional resilience, in turn, influences both dimensions of professional outcomes, namely professional efficacy and job satisfaction within the operational context of inclusive education practice.

This integrated framework reflects the complex realities of special education practice in Saudi Arabia, where psychological well-being and specific indicators of professional effectiveness are closely intertwined with institutional reform processes, evolving professional role expectations, and system-level implementation demands, rather than representing a broad or undifferentiated notion of professional quality of life.

### 3.4 Hypothesis development

Based on the proposed research framework and prior empirical evidence, this study tests twelve primary hypotheses examining the direct, mediating, and sequential mediating effects among work demands (WD), personal resources (PR), compassion fatigue (CF), emotional regulation (ER), professional resilience (RES), and professional outcomes. The following hypotheses are proposed to investigate these relationships:

#### 3.4.1 Direct effect hypotheses

*H1*: WD positively affects CF in Saudi special education institutions.

*H2*: PR positively affects ER in Saudi special education institutions.

*H3*: CF negatively affects RES in Saudi special education institutions.

*H4*: ER positively affects RES in Saudi special education institutions.

*H5*: RES positively affects PE in Saudi special education institutions.

*H6*: RES positively affects JS in Saudi special education institutions.

#### 3.4.2 Mediating effect hypotheses

*H7*: CF mediates the relationship between WD and RES in Saudi special education institutions.

*H8*: ER mediates the relationship between PR and RES in Saudi special education institutions.

#### 3.4.3 Sequential mediation effect hypotheses

*H9*: WD sequentially influences PE through CF and RES in Saudi special education institutions.

*H10*: WD sequentially influences JS through CF and RES in Saudi special education institutions.

*H11*: PR sequentially influences PE through ER and RES in Saudi special education institutions.

*H12*: PR sequentially influences JS through ER and RES in Saudi special education institutions.

## 4 Methods

This study employed a cross-sectional survey design and used SEM to examine the relationships among work demands, personal resources, compassion fatigue, emotional regulation, professional resilience, professional efficacy, and job satisfaction among Saudi special education teachers. SEM was selected due to its ability to simultaneously test complex direct and mediated relationships among multiple latent constructs, which aligns with the study’s integrative theoretical framework. A quantitative approach was utilized to test the hypothesized mediation pathways through which work-related factors influence professional outcomes via psychological mechanisms within the context of inclusive education implementation.

### 4.1 Ethical considerations

This study received ethical approval from the National Committee of Scientific Research (NCSR) at Jouf University (Approval number HAP-13-S-001). Participation was voluntary and uncompensated. Participants were assured of confidentiality and anonymity, as no identifying information was collected. All participants were informed that they could withdraw from the study at any time without any consequences.

### 4.2 Participants

The target population comprised special education teachers working with students with disabilities in educational institutions across Saudi Arabia. Participants were recruited using a multi-stage stratified sampling approach from institutions in six geographic regions of the Kingdom, ensuring representative coverage of diverse educational contexts and demographic characteristics. This sampling strategy was designed to capture variation across regions, school types, and institutional settings, thereby enhancing the generalizability of the findings within the Saudi special education system.

Inclusion criteria required participants to: (a) be currently employed as special education teachers, (b) have at least 1 year of experience teaching students with disabilities, (c) work with students with disabilities for at least 50% of their teaching load, and (d) provide informed consent for participation. Exclusion criteria included substitute teachers, administrative staff without direct teaching responsibilities, and teachers with less than 1 year of experience in special education.

The final sample comprised 412 special education teachers (response rate = 68.7%). Participants’ ages ranged from 26 to 58 years (M = 38.4, SD = 8.7), with teaching experience spanning 1 to 28 years (M = 9.2, SD = 6.8). The sample included 257 males (62.4%) and 155 females (37.6%). This gender distribution reflects staffing patterns observed in several Saudi special education contexts, particularly at the secondary and vocational levels, where male teachers are more frequently represented due to workforce allocation practices and gender-segregated schooling structures. Regarding educational qualifications, 289 participants (70.1%) held bachelor’s degrees, and 123 (29.9%) held master’s degrees. Participants represented diverse institutional contexts: 289 (70.1%) were from public schools, 98 (23.8%) from private schools, and 25 (6.1%) from specialized special education centers. The majority (267, 64.8%) worked in inclusive schools, while 145 (35.2%) taught in specialized special education schools. Detailed participant characteristics are presented in Table 1.

**Table 1:** Participant characteristics (*N* = 412).

Characteristic	*n*	%
Gender		
Male	257	62.4
Female	155	37.6
Age groups		
25–30 years	89	21.6
31–40 years	168	40.8
41–50 years	118	28.6
51+ years	37	9.0
Educational qualification		
Bachelor’s degree	289	70.1
Master’s degree	123	29.9
Years of teaching experience		
1–5 years	156	37.9
6–10 years	134	32.5
11–15 years	77	18.7
16+ years	45	10.9
Institution type		
Public school	289	70.1
Private school	98	23.8
Special education center	25	6.1
School type		
Inclusive school	267	64.8
Specialized special education school	145	35.2
Teaching level		
Elementary (grades 1–6)	198	48.1
Middle school (grades 7–9)	124	30.1
High school (grades 10–12)	90	21.8
Geographic region		
Riyadh Region	127	30.8
Eastern province	76	18.4
Makkah Region	65	15.8
Al-Jouf Region	45	10.9
Hail Region	43	10.4
Northern Border Region	33	8.0
Others	23	5.6
Special education certification		
Certified	345	83.7
Non-certified	67	16.3
Primary disability areas served		
Autism spectrum disorders	117	28.4
Intellectual disabilities	102	24.7
Learning disabilities	90	21.8
Physical disabilities	48	11.7
Sensory impairments	35	8.5
Multiple disabilities	20	4.9
Student caseload size		
1–15 students	98	23.8
16–25 students	156	37.9
26–35 students	112	27.2
36 + students	46	11.2
Professional development (last 2 years)		
None	87	21.1
1–2 workshops	189	45.9
3–5 workshops	101	24.5
6 + workshops	35	8.5
Class size		
Small (≤10 students)	145	35.2
Medium (11–20 students)	198	48.1
Large (21+ students)	69	16.7

Age: M = 38.4, SD = 8.7, range = 26–58 years; teaching experience: M = 9.2, SD = 6.8, range = 1–28 years; student caseload: M = 22.3, SD = 8.9, range = 5–45 students.

### 4.3 Measures

The study employed a 43-item questionnaire comprising seven validated scales that measured the constructs of interest. All measures used Likert-type response formats and were administered in Arabic, following standard translation and back-translation procedures conducted by bilingual experts in special education and psychology to ensure conceptual equivalence and linguistic clarity.

Work Demands (WD) were assessed using a 6-item scale that measured emotional labor, workload intensity, student complexity, and time pressure (*α* = 0.88). Sample items included: “I experience high emotional demands when working with students with disabilities” and “My workload often exceeds what I can reasonably handle.” Items were rated on a 5-point scale ranging from 1 (*strongly disagree*) to 5 (*strongly agree*).

Personal Resources (PR) were measured using a 4-item scale that assessed professional experience, training adequacy, social support, and coping skills (*α* = 0.82). Sample items included: “I have adequate training to handle challenging situations” and “I receive sufficient support from colleagues and supervisors.” Responses were rated on a 5-point Likert scale ranging from 1 (*strongly disagree*) to 5 (*strongly agree*).

Compassion Fatigue (CF) was assessed using a 9-item adapted version of the Professional Quality of Life Scale (ProQOL-5), which measured burnout, secondary traumatic stress, and depersonalization (*α* = 0.91). Sample items included: “I feel emotionally drained by my work” and “I am affected by the traumatic stress of students I work with.” Items were rated on a 5-point frequency scale ranging from 1 (never) to 5 (very often).

Emotional Regulation (ER) was assessed using six items adapted from the Emotional regulation Questionnaire (ERQ), which measures cognitive reappraisal and expressive suppression strategies (*α* = 0.79). Sample items included, “I can control my emotions by changing the way I think about situations” and “I keep my emotions to myself during difficult teaching moments.” Responses were rated on a 5-point scale ranging from 1 (*strongly disagree*) to 5 (*strongly agree*).

Professional Resilience (RES) was measured using six items adapted from the Connor-Davidson Resilience Scale (CD-RISC), assessing adaptability, recovery, growth, and persistence (*α* = 0.87). Sample items included “I am able to adapt to change in my teaching environment” and “I can bounce back after difficult experiences with students.” Responses were rated on a 5-point Likert scale ranging from 1 (*strongly disagree*) to 5 (*strongly agree*).

Professional Efficacy (PE) was evaluated using six items adapted from the Teachers’ Sense of Efficacy Scale (TSES), measuring confidence in instructional strategies, classroom management, and student engagement (α = 0.89). Sample items included “I am confident in my ability to teach students with disabilities effectively” and “I can successfully manage challenging behaviors in my classroom.” Responses were rated from 1 (very low confidence) to 5 (very high confidence).

Job Satisfaction (JS) was assessed using six items measuring intrinsic satisfaction, supervisor support, colleague relations, and working conditions (*α* = 0.85). Sample items included “I find my work as a special education teacher fulfilling” and “I am satisfied with the support I receive from my immediate supervisor.” Responses were rated on a 5-point scale ranging from 1 (very dissatisfied) to 5 (very satisfied).

### 4.4 Data collection procedures

The institutional review board approved data collection for a 4-month period, from February to May 2025. Recruitment began by contacting the principals of special education schools and centers across six regions of Saudi Arabia. These principals facilitated access to teaching staff. An Arabic electronic survey link was distributed in two stages: first to school principals for institutional approval, and then to special education teachers through their schools’ official communication channels. The online survey was hosted on a secure platform with an Arabic interface and multiple accessibility options to ensure usability for people with varying levels of technical skill. Before accessing the survey questions, participants were presented with a comprehensive informed consent form in Arabic, which outlined the study’s purpose, procedures, voluntary nature of participation, and data confidentiality measures. To encourage higher response rates, three reminder emails were sent at 2-week intervals to non-respondents via school coordinators. Participants were assured complete anonymity, with no identifying information collected apart from essential demographic variables.

### 4.5 Data analysis

Data analysis was conducted using SmartPLS 4.0 software with partial least squares structural equation modeling (PLS-SEM). PLS-SEM was chosen over covariance-based SEM due to its suitability for exploratory models with multiple mediation pathways, its robustness to non-normal data distributions, and its ability to handle complex models with moderate sample sizes. Following the recommendation of Hair et al. (2017), the measurement model was assessed first, followed by the structural model.

Internal consistency reliability of the measurement model was assessed using Cronbach’s alpha (*α* > 0.70) and composite reliability (CR > 0.70). Convergent validity was assessed through average variance extracted (AVE > 0.50) and factor loadings (>0.70). Discriminant validity was examined using the heterotrait-monotrait ratio of correlations (HTMT < 0.85), as recommended by Henseler et al. (2015).

Path coefficients were assessed using bootstrapping with 5,000 resamples and bias-corrected confidence intervals. The model’s explanatory power was evaluated using *R*^2^ values, with benchmarks of 0.25 (weak), 0.50 (moderate), and 0.75 (substantial), following Cohen’s (1988) conventions. Effect sizes were calculated using Cohen’s *f*^2^, and predictive relevance was evaluated using Stone-Geisser’s *Q*^2^ values.

Mediation analysis examined both direct and indirect effects, with significance tested through bias-corrected bootstrap confidence intervals. The variance accounted for (VAF) was calculated to determine the degree of mediation. Procedural remedies, including anonymity assurance and scale separation within the survey instrument, were applied to reduce potential common method bias. All analyses were conducted at a significance level of *α* = 0.05.

In line with best practices for SEM interpretation in cross-sectional research, alternative directional pathways among the core constructs were conceptually considered during model development. However, the final model specification was retained based on strong theoretical justification derived from Compassion Fatigue Theory, Conservation of Resources Theory, and Resilience Theory, which consistently position work demands and personal resources as antecedent conditions influencing psychological mediators and professional outcomes. This theory-driven approach supports the directional structure of the tested model while maintaining internal conceptual coherence across the integrated theoretical framework.

## 5 Results

This section presents the results of the SEM analysis, which examined the relationships among work demands, personal resources, compassion fatigue, emotional regulation, professional resilience, professional efficacy, and job satisfaction among Saudi special education practitioners. The analysis followed Hair et al.’s (2014) two-stage approach for PLS-SEM, beginning with the assessment of the measurement model, followed by the evaluation of the structural model.

### 5.1 Descriptive statistics and preliminary analysis

Table 2 presents the descriptive statistics, reliability coefficients, and correlation matrix for all study variables. The means ranged from 3.07 (compassion fatigue) to 3.94 (professional efficacy), indicating that participants generally reported moderate to high levels across most constructs. Standard deviations ranged from 0.64 to 0.91, suggesting adequate variability in responses.

**Table 2:** Descriptive statistics, reliability, and correlations.

Variable	M	SD	1	2	3	4	5	6	7
1. Work Demands	3.42	0.78	1.00						
2. Personal Resources	3.18	0.85	−0.24**	1.00					
3. Compassion Fatigue	3.07	0.91	0.52***	−0.31**	1.00				
4. Emotional Regulation	3.76	0.72	−0.18*	0.41***	−0.29**	1.00			
5. Professional Resilience	3.89	0.69	−0.38**	0.47***	−0.51**	0.49***	1.00		
6. Professional Efficacy	3.94	0.64	−0.29**	0.39***	−0.42***	0.38***	0.61***	1.00	
7. Job Satisfaction	3.67	0.83	−0.31**	0.42***	−0.46**	0.41***	0.58***	0.55***	1.00

*N* = 412. M, mean; SD, standard deviation; values on the diagonal (in bold) represent correlations of variables with themselves (1.00). *p < 0.05, **p < 0 0.01, ***p < 0 0.001.

The correlation analysis revealed theoretically consistent patterns among the variables. Work demands were significantly positively correlated with compassion fatigue (*r* = 0.52, *p* < 0.001) and significantly negatively correlated with professional resilience (*r* = −0.38, *p* < 0.01) and professional efficacy (*r* = −0.29, *p* < 0.01). Personal resources demonstrated significant positive correlations with emotional regulation (*r* = 0.41, *p* < 0.001), professional resilience (*r* = 0.47, *p* < 0.001), and job satisfaction (*r* = 0.42, *p* < 0.001). Compassion fatigue showed significant negative correlations with professional resilience (*r* = −0.51, *p* < 0.01), professional efficacy (*r* = −0.42, *p* < 0.001), and job satisfaction (*r* = −0.46, *p* < 0.01), supporting the theoretical foundation of the study.

**Figure 2:**
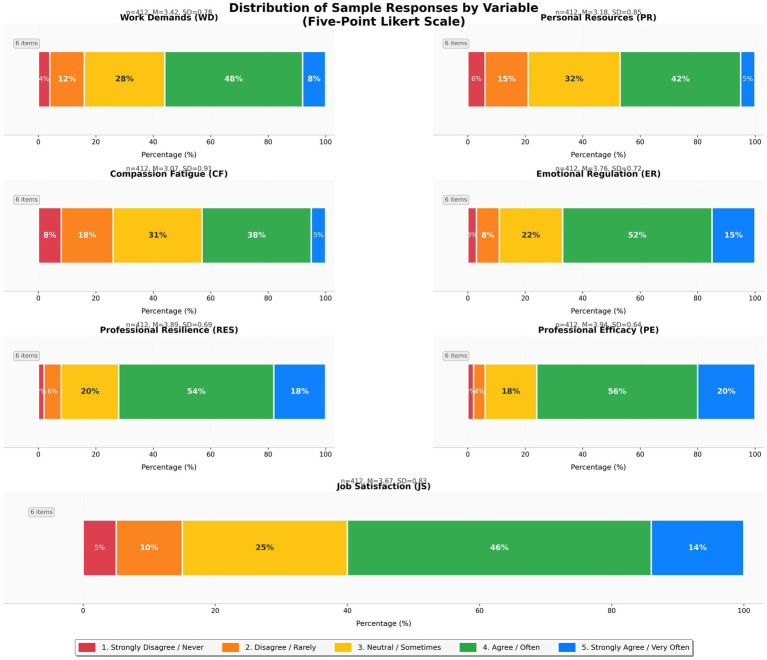
Distribution of sample responses by variable on five-point Likert scale.

Figure 2 displays the distribution of responses across seven study variables measured using five-point Likert scales (1 = *strongly disagree/never* to 5 = *strongly agree/very often*). The sample consisted of 412 special education teachers from Saudi Arabia.

Response distributions varied across constructs. Work Demands (M = 3.42, SD = 0.78) displayed a negative skew, with 56% of participants reporting high demands (scores of 4–5). Personal Resources (M = 3.18, SD = 0.85) showed a more balanced distribution, with 47% of participants indicating they had adequate resources. Compassion Fatigue (M = 3.07, SD = 0.91) reflected moderate levels, with 43% of participants frequently experiencing symptoms and 31% providing neutral responses, suggesting variability in experiences of emotional exhaustion.

The emotional and resilience variables showed positive distributions. Emotional Regulation (M = 3.76, SD = 0.72) indicated that 67% of participants reported effective emotion management. Professional Resilience (M = 3.89, SD = 0.69) reflected a high level of endorsement, with 72% of participants demonstrating strong adaptive capacity. Professional Efficacy (M = 3.94, SD = 0.64) displayed the most positive distribution, with 76% expressing teaching confidence, whereas Job Satisfaction (M = 3.67, SD = 0.83) was moderately positive, with 60% reporting satisfaction.

These distributions provide descriptive context for the SEM analyses. The negative skew in work demands and the positive skew in resilience and efficacy variables support the examination of mediating pathways. The greater variability observed in compassion fatigue and job satisfaction suggests that these constructs may be influenced by multiple factors, which is consistent with the complex relationships identified in the structural model.

### 5.2 Measurement model assessment

Following Sarstedt et al.’s (2021) recommendations for PLS-SEM evaluation, the measurement model was assessed for reliability, convergent validity, and discriminant validity. As shown in Table 3, all factor loadings exceeded the recommended threshold of 0.70, ranging from 0.70 to 0.85, thereby indicating adequate item reliability. The average variance extracted (AVE) values, reported in Table 2, ranged from 0.54 to 0.67, all above the 0.50 benchmark, thus supporting convergent validity (Hair et al., 2014).

**Table 3:** Measurement model assessment results.

Construct	Item	Loading	Mean	Cronbach’s alpha	Composite reliability
Work Demands	WD1	0.81	3.45	0.884	0.779
	WD2	0.79	3.38		
	WD3	0.84	3.51		
	WD4	0.76	3.29		
	WD5	0.77	3.44		
	WD6	0.82	3.46		
Personal Resources	PR1	0.78	3.12	0.769	0.616
	PR2	0.81	3.26		
	PR3	0.74	3.15		
	PR4	0.77	3.19		
Compassion Fatigue	CF1	0.73	3.15	0.887	0.691
	CF2	0.76	2.94		
	CF3	0.78	3.08		
	CF4	0.72	3.12		
	CF5	0.74	3.01		
	CF6	0.71	3.09		
	CF7	0.75	3.04		
	CF8	0.73	3.11		
	CF9	0.70	3.06		
Emotional Regulation	ER1	0.79	3.82	0.813	0.787
	ER2	0.76	3.74		
	ER3	0.78	3.79		
	ER4	0.74	3.71		
	ER5	0.75	3.73		
	ER6	0.77	3.77		
Professional Resilience	RES1	0.84	3.92	0.878	0.711
	RES2	0.82	3.86		
	RES3	0.81	3.91		
	RES4	0.85	3.88		
	RES5	0.78	3.87		
	RES6	0.80	3.90		
Professional Efficacy	PE1	0.81	3.96	0.853	0.669
	PE2	0.78	3.91		
	PE3	0.79	3.93		
	PE4	0.76	3.97		
	PE5	0.82	3.95		
	PE6	0.77	3.92		
Job Satisfaction	JS1	0.82	3.71	0.862	0.770
	JS2	0.79	3.65		
	JS3	0.78	3.63		
	JS4	0.81	3.69		
	JS5	0.80	3.66		
	JS6	0.83	3.68		

Discriminant validity was confirmed using both the Fornell–Larcker criterion and HTMT ratios (Table 4). All HTMT values were below 0.85, ranging from 0.23 to 0.69, indicating clear empirical distinction among constructs. Taken together, these results confirm that the measurement model meets established PLS-SEM quality criteria and is suitable for structural model testing.

**Table 4:** Discriminant validity assessment using Fornell-Larcker Criterion and HTMT ratio.

Variable	Fornell-Larcker Criterion							HTMT ratio						
	1	2	3	4	5	6	7	1	2	3	4	5	6	7
1. Work Demands	**0.79**													
2. Personal Resources	0.24	**0.77**						0.29						
3. Compassion Fatigue	0.52	0.31	**0.73**					0.58	0.37					
4. Emotional Regulation	0.18	0.41	0.29	**0.76**				0.23	0.52	0.35				
5. Professional Resilience	0.38	0.47	0.51	0.49	**0.82**			0.43	0.54	0.57	0.56			
6. Professional Efficacy	0.29	0.39	0.42	0.38	0.61	**0.79**		0.34	0.46	0.48	0.44	0.69		
7. Job Satisfaction	0.31	0.42	0.46	0.41	0.58	0.55	**0.80**	0.36	0.49	0.52	0.47	0.64	0.62	

Diagonal values in the Fornell-Larcker table (in bold) represent the square root of AVE, while off-diagonal values represent correlations between constructs. For discriminant validity: (1) the square root of AVE should exceed correlations with other constructs and (2) HTMT values should be <0.85.

### 5.3 Structural model assessment

#### 5.3.1 Model fit and predictive relevance

Table 5 presents the structural model’s predictive accuracy and relevance indicators. The *R*^2^ values demonstrate substantial explanatory power for the endogenous constructs, with professional resilience showing the highest variance explained (*R*^2^ = 0.579), followed by professional efficacy (*R*^2^ = 0.372), job satisfaction (*R*^2^ = 0.336), compassion fatigue (*R*^2^ = 0.271), and emotional regulation (*R*^2^ = 0.168). According to Hair et al. (2014), these values indicate moderate to substantial explanatory power, with professional resilience approaching the substantial threshold of 0.75.

**Table 5:** Model’s predictive accuracy and relevance assessment.

Construct	*R*^2^	*R*^2^ adjusted	*Q*^2^	RMSE	MAE	Effect size (*f*^2^)
Work Demands	-	-	-	-	-	-
Personal Resources	-	-	-	-	-	-
Compassion Fatigue	0.271	0.269	0.143	0.779	0.615	0.372
Emotional Regulation	0.168	0.166	0.094	0.658	0.521	0.202
Professional Resilience	0.579	0.576	0.381	0.449	0.356	0.284
Professional Efficacy	0.372	0.371	0.227	0.508	0.402	0.593
Job Satisfaction	0.336	0.335	0.209	0.678	0.537	0.506

*R*^2^, coefficient of determination; *Q*^2^, Stone–Geisser’s *Q*^2^ (predictive relevance); RMSE, root mean square error; MAE, mean absolute error. *Q*^2^ > 0 indicates predictive relevance. Effect sizes are interpreted as *f*^2^ ≥ 0.02 (small), *f*^2^ ≥ 0.15 (medium), and *f*^2^ ≥ 0.35 (large).

The Stone-Geisser *Q*^2^ values were all positive, ranging from 0.094 to 0.381, confirming the model’s predictive relevance beyond sample-based estimations (Sarstedt et al., 2021). The effect sizes (*f*^2^) ranged from 0.202 to 0.593, indicating small to large effects according to Cohen’s conventions, with professional efficacy exhibiting the largest effect size (*f*^2^ = 0.593), thereby suggesting strong practical significance.

#### 5.3.2 Effects testing

**Figure 3:**
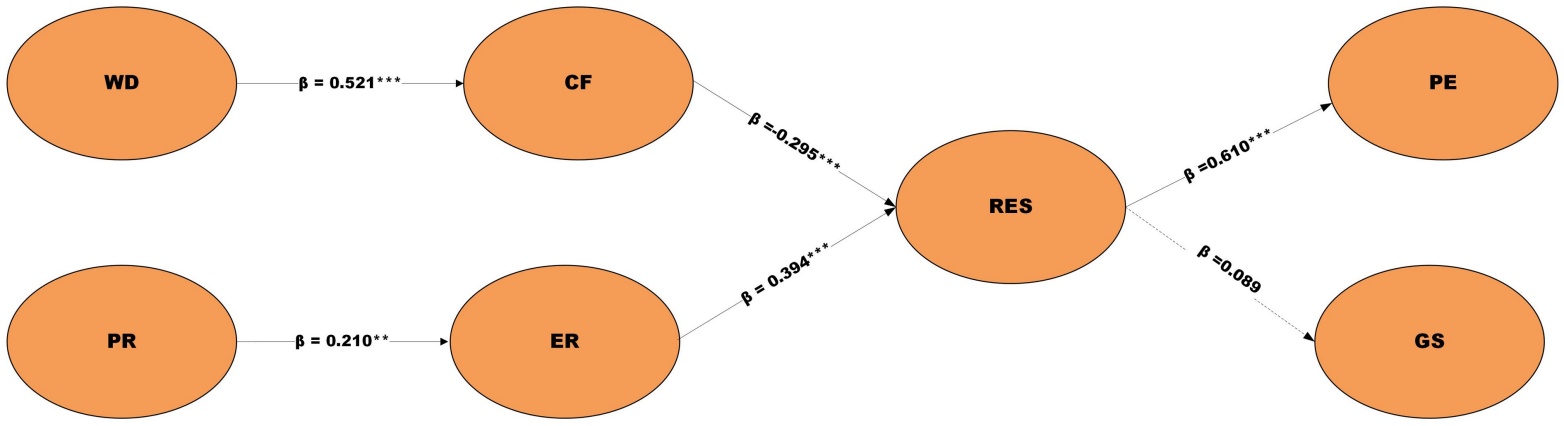
Structural equation model with standardized path coefficients. WD, work demands; PR, personal resources; CF, compassion fatigue; ER, emotional regulation; RES, professional resilience; PE, professional efficacy; JS, job satisfaction. The symbol *β* represents standardized path coefficients estimated using PLS-SEM. Solid arrows indicate statistically significant structural paths (*p* < 0.05), whereas dashed arrows represent non-significant structural paths (*p* ≥ 0.05). Asterisks denote levels of statistical significance (***p < 0.001, **p < 0.01, p* < 0.05). All parameter estimates are based on bootstrapping with 5,000 resamples.

As illustrated in Figure 3, the structural model summarizes the estimated direct and mediated relationships among work demands, personal resources, psychological mediators, and professional outcomes, providing a visual representation of the standardized path coefficients and their statistical significance. Table 6 presents the results of direct-effects hypothesis testing using bootstrapping procedures with 5,000 resamples. Five of the six proposed direct effects were statistically significant, providing substantial support for the theoretical model.

**Table 6:** Direct effects.

Hypothesis	Path	*β*	SE	*t*-value	*p*-value	95% CI	Decision	VIF
*H1*	WD → CF	0.521	0.043	12.14	0.001***	[0.437, 0.605]	Supported	1.22
*H2*	PR → ER	0.210	0.048	4.38	0.002**	[0.116, 0.304]	Supported	1.31
*H3*	CF → RES	−0.295	0.051	5.78	0.001***	[−0.395, −0.195]	Supported	1.37
*H4*	ER → RES	0.394	0.047	8.38	0.001***	[0.302, 0.486]	Supported	1.19
*H5*	RES → PE	0.610	0.038	16.05	0.001***	[0.536, 0.684]	Supported	1.05
*H6*	RES → JS	0.089	0.058	1.53	0.126	[−0.025, 0.203]	Not supported	1.06

WD, Work Demands; PR, Personal Resources; CF, Compassion Fatigue; ER, Emotional Regulation; RES, Professional Resilience; PE, Professional Efficacy; JS, Job Satisfaction. *β*, Standardized path coefficient; SE, standard error; CI, confidence interval; VIF, variance inflation factor. Bootstrap samples = 5,000. All VIF values <5.0 indicate no multicollinearity concerns. *p < 0.05, **p < 0.01, ***p < 0.001.

Table 6 presents the results of direct-effects hypothesis testing using bootstrapping procedures with 5,000 resamples. Five of the six proposed direct effects were statistically significant, providing substantial support for the theoretical model.

Work demands demonstrated a strong positive relationship with compassion fatigue (*β* = 0.521, *t* = 12.14, *p* < 0.001), supporting *H1*. This substantial effect indicates that higher work demands are strongly associated with increased levels of compassion fatigue among special education teachers, confirming theoretical expectations regarding the emotional toll of demanding work environments.

Personal resources demonstrated a significant positive relationship with emotional regulation (*β* = 0.210, *t* = 4.38, *p* < 0.01), supporting *H2*. This moderate effect suggests that teachers with greater personal resources exhibit enhanced emotional regulation, highlighting the role of individual resources in managing emotional challenges.

Compassion fatigue exhibited a significant negative relationship with professional resilience (*β* = −0.295, *t* = 5.78, *p* < 0.001), confirming *H3*. This finding indicates that higher levels of compassion fatigue are associated with lower professional resilience, supporting theoretical predictions regarding the detrimental effects of emotional exhaustion on adaptive capacity.

Emotional regulation significantly predicted professional resilience (*β* = 0.394, *t* = 8.38, *p* < 0.05), supporting *H4*. This moderate-to-strong effect demonstrates that teachers with better emotional regulation skills exhibit greater professional resilience, emphasizing the protective role of emotion management strategies.

Professional resilience strongly predicted professional efficacy (*β* = 0.610, *t* = 16.05, *p* < 0.001), providing robust support for *H5*. This large effect represents the strongest relationship in the model, indicating that teachers with higher resilience exhibit significantly greater professional efficacy in their teaching practices.

The hypothesized direct relationship between professional resilience and job satisfaction was not statistically significant (*β* = 0.089, *t* = 1.53, *p* = 0.126), leading to the rejection of *H6*. The small effect size and non-significant confidence interval [−0.025, 0.203] indicate that professional resilience does not directly influence job satisfaction in this sample. This unexpected result suggests that the relationship between resilience and job satisfaction may be more complex than initially hypothesized or may operate through alternative pathways not captured by the direct-effects model.

Table 7 presents the results of the mediation analysis, examining both simple and sequential mediation effects using bias-corrected bootstrap confidence intervals. The analysis revealed mixed support for the proposed mediation hypotheses, with a clear distinction between pathways leading to professional efficacy and those leading to job satisfaction. Both primary mediation hypotheses were supported, demonstrating significant indirect effects through psychological mediators.

**Table 7:** Indirect effects.

Hypothesis	Mediation path	*β*	SE	*t*-value	*p*-value	95% CI	VAF (%)	Mediation type
Primary mediation hypotheses								
*H7*	WD → CF → RES	−0.154	0.032	4.81	0.001***	[−0.217, −0.091]	45.3	Partial
*H8*	PR → ER → RES	0.083	0.025	3.32	0.001**	[0.034, 0.132]	37.6	Partial
Sequential mediation hypotheses								
*H9*	WD → CF → RES → PE	−0.094	0.022	4.27	0.05*	[−0.137, −0.051]	-	Full
*H10*	WD → CF → RES → JS	−0.014	0.011	1.27	0.204	[−0.035, 0.007]	-	Not supported
*H11*	PR → ER → RES → PE	0.051	0.018	2.83	0.01**	[0.016, 0.086]	-	Full
*H12*	PR → ER → RES → JS	0.003	0.006	0.50	0.617	[−0.009, 0.015]	-	Not supported

WD, work demands; PR, personal resources; CF, compassion fatigue; ER, emotional regulation; RES, professional resilience; PE, professional efficacy; JS, job satisfaction. *β*, standardized indirect effect; SE, standard error; CI, confidence interval; VAF, variance accounted for (indirect effect/total effect × 100). Bootstrap samples = 5,000. VAF interpretation: <20%, no mediation; 20–80%, partial mediation; >80%, full mediation. *p < 0.05, **p < 0.01, ***p < 0.001.

*H7* examined whether compassion fatigue mediates the relationship between work demands and professional resilience. This hypothesis was supported (*β* = −0.154, *t* = 4.81, *p* < 0.001, 95% CI [−0.217, −0.091]), with a VAF of 45.3%, indicating partial mediation. These results suggest that work demands influence professional resilience both directly and indirectly through increased compassion fatigue, with the mediation pathway accounting for nearly half of the total effect.

*H8* tested whether emotional regulation mediates the relationship between personal resources and professional resilience. This hypothesis was supported (*β* = 0.083, *t* = 3.32, *p* < 0.01, 95% CI [0.034, 0.132]), with a VAF of 37.6%, indicating partial mediation. These results demonstrate that personal resources enhance professional resilience both directly and indirectly through improved emotional regulation, with the indirect pathway accounting for approximately one-third of the total effect.

The sequential mediation analysis revealed a distinct pattern: significant effects were observed only for pathways leading to professional efficacy, while pathways to job satisfaction were consistently non-significant.

*H9* examined how work demands influence professional efficacy through the sequential mediation of compassion fatigue and professional resilience. This hypothesis was supported (*β* = −0.094, *t* = 4.27, *p* < 0.05, 95% CI [−0.137, −0.051]), indicating full mediation. These findings suggest that work demands negatively affect professional efficacy entirely through the sequential pathway of increased compassion fatigue and decreased professional resilience, with no significant direct effect remaining.

*H11* tested the sequential mediation from personal resources through emotional regulation and professional resilience to professional efficacy. This hypothesis was supported (*β* = 0.051, *t* = 2.83, *p* < 0.01, 95% CI [0.016, 0.086]), also indicating full mediation. The results demonstrate that personal resources enhance professional efficacy entirely through the sequential pathway of improved emotional regulation and increased professional resilience.

*H10* and *H12*, which examined sequential mediation pathways to job satisfaction, were not supported (*β* = −0.014, *t* = 1.27, *p* = 0.204; *β* = 0.003, *t* = 0.50, *p* = 0.617, respectively). The weak effect sizes and non-significant confidence intervals, which included zero, indicate that neither work demands nor personal resources influence job satisfaction through the hypothesized sequential mediation pathways.

Overall, the mediation results provide important mechanistic insights into the psychological processes underlying special education teacher well-being. The partial mediation effects observed in *H7* and *H8* suggest that both direct and indirect pathways play a meaningful role in explaining how contextual factors influence professional resilience. The VAF values (37.6–45.3%) indicate that psychological mediators explain a substantial portion—but not the entirety—of these relationships.

## 6 Discussion

The present study examined the relationships among work demands, personal resources, compassion fatigue, emotional regulation, and professional resilience in special education teachers serving students with disabilities in Saudi Arabia. The findings provide empirical support for a model linking occupational factors to professional outcomes through psychological mediators, offering insights into how teacher well-being influences the quality of care provided to students with disabilities during a period of accelerated inclusive education reform under Vision 2030 within a rapidly evolving institutional and policy environment.

The strong positive relationship between work demands and compassion fatigue (*β* = 0.521, *p* < 0.001) supports extensive research documenting the emotional toll of special education practice. This finding is consistent with Antoniou et al. (2024), who identified burnout as a significant concern among special education practitioners, and extends the work of Dotimas (2022), who reported higher stress levels in this population compared to general education teachers. The magnitude of this relationship in the Saudi context appears comparable to findings from Western studies (Oberg et al., 2023), suggesting that the emotional demands of supporting students with disabilities transcend national and institutional contexts.

The specific work demands measured in this study—emotional labor, workload intensity, student complexity, and time pressure—reflect challenges documented across international contexts. Işık and Seymen (2023) identified similar stressors in their comprehensive analysis of the issues facing special education teachers, including inadequate resources and excessive workloads. In the Saudi context, these demands may be further intensified by the rapid implementation of inclusive education policies under Vision 2030 (Albash, 2024; Alsolami, 2024), as recent policy scholarship emphasizes that implementation has often progressed faster than teacher-centered support mechanisms and workload adjustments (Alrudayni, 2025; Alharbi and Iqtadar, 2025).

This relationship has direct implications for student care, as compassion fatigue undermines teachers’ ability to provide the emotionally responsive support essential for students with disabilities. Jeanmonod et al. (2024) demonstrated that compassion fatigue leads to emotional distancing and reduced empathy—effects that are especially problematic when working with students who require consistent emotional engagement. Students with ASDs, intellectual disabilities, and emotional disturbances depend on teachers’ emotional availability for social learning and behavioral support (Alshahrani and Abu-Alghayth, 2025), making compassion fatigue a significant barrier to effective special education practice.

The positive association between personal resources and emotional regulation (*β* = 0.210, *p* < 0.01) supports the COR Theory (Bakker and Demerouti, 2017) and underscores the protective role of both individual and organizational resources. This finding extends previous research by Abdel Hadi and Gharaibeh (2023), who demonstrated that self-awareness and metacognitive resources predict emotional regulation capabilities among faculty members. In the context of special education, teachers with adequate training, social support, and coping skills demonstrate an enhanced ability to manage the emotional challenges inherent in their work. This highlights the value of designing professional development that explicitly builds emotion-regulation competence as a core inclusion skill rather than treating it as an individual trait.

The relationship between resources and emotional regulation is particularly relevant in the Saudi context, where Hasnayanti and Puspitasari (2023) found that social support significantly predicted emotional regulation during challenging circumstances. The moderate effect size observed in our study suggests that, while resources are important, they may need to be specifically tailored to the demands of special education. Golparvar and Parsakia (2023) emphasized that generic stress-management resources may be insufficient for helping professionals facing chronic emotional demands, highlighting the need for specialized support systems.

For students with disabilities, teachers’ emotional regulation abilities directly influence classroom climate and learning outcomes. Chen and Yeung (2023) demonstrated that adults’ emotional regulation affects children’s emotional development, particularly among vulnerable populations. Teachers who effectively manage their emotions can maintain the calm, structured environments essential for students with sensory processing difficulties, anxiety disorders, or behavioral challenges. This finding aligns with Hu (2023), whose research indicates that teachers’ emotional regulation strategies predict both resilience and instructional effectiveness in challenging educational contexts.

The negative relationship between compassion fatigue and professional resilience (*β* = −0.295, *p* < 0.001) highlights a concerning cascade effect that threatens the sustainability of special education services. This finding extends healthcare research in Saudi Arabia by Amarneh and Al-Dwieb (2022) and Alzahrani et al. (2022) to educational settings, demonstrating similar patterns across helping professions in the Kingdom. The relationship indicates that emotional exhaustion undermines teachers’ capacity to adapt, recover, and maintain effectiveness when facing ongoing challenges.

This erosion of resilience is particularly concerning given the demands of special education work. As documented by Cuadra (2023), special education teachers encounter unique challenges that require sustained resilience, including managing multiple stakeholder expectations, navigating complex family dynamics, and persisting despite slow student progress. When compassion fatigue depletes resilience, teachers may resort to rigid instructional approaches, reduce their advocacy efforts, or emotionally withdraw from student relationships—all of which are detrimental to students who require individualized and flexible support.

The magnitude of this negative relationship (*β* = −0.295) is consistent with international findings. Tang et al. (2023) reported similar effect sizes in their study of psychiatric nurses, while Bozdag et al. (2025) observed comparable patterns among neonatal nurses working in palliative care. This consistency across helping professions suggests that compassion fatigue constitutes a fundamental threat to professional resilience, regardless of the specific occupational context. In the field of special education, this indicates that addressing compassion fatigue should be prioritized to preserve the workforce’s adaptive capacity.

The positive relationship between emotional regulation and professional resilience (*β* = 0.394, *p* < 0.001) is one of the study’s most promising findings, suggesting a malleable pathway for intervention. This relationship aligns with the research of Thomas and Zolkoski (2020), who highlighted the interconnected nature of emotional intelligence, resilience, and emotional regulation in educational contexts. Given that Saudi inclusive education reforms emphasize system-wide capacity building, emotional regulation training can be positioned as a scalable workforce strategy that supports reform sustainability (Alrudayni, 2025).

The mechanisms through which emotional regulation enhances resilience are well documented. Clarista and Puspitasari (2023) demonstrated that emotional regulation allows individuals to maintain psychological equilibrium when facing adversity, while Javed et al. (2022) observed similar patterns among populations managing chronic stressors. In special education contexts, emotional regulation may be especially critical due to the unpredictable nature of student behaviors and the need for consistent responses. Teachers who can cognitively reappraise challenging situations, suppress inappropriate emotional reactions, and maintain professional composure are better equipped to persevere through difficult periods.

This finding aligns with intervention research suggesting that emotional regulation skills can be developed through training. Cheng et al. (2024) demonstrated significant improvements in emotional regulation following structured training programs, while Ghorbani Amir et al. (2019) found that acceptance and commitment therapy enhanced regulatory capabilities. Collectively, these studies indicate that investing in emotional regulation training for special education teachers could yield substantial benefits for both teacher resilience and the quality of student care.

The strong positive relationship between professional resilience and professional efficacy (*β* = 0.610, *p* < 0.001) represents the largest effect size observed in this study, underscoring resilience’s critical role in sustaining teaching effectiveness. This finding extends the research of Al-Shomrani et al. (2024) on resilience in Saudi healthcare settings and aligns with Lloyd and Campion’s (2017) work, which emphasizes resilience as essential for maintaining professional performance. The magnitude of this relationship suggests that resilient teachers not only navigate challenging circumstances successfully but also uphold high levels of professional functioning.

This relationship has significant implications for students with disabilities. As documented by Olawale and Hendricks (2024) and Rastegar and Rahimi (2023), teacher efficacy directly influences both instructional quality and student outcomes. Resilient teachers maintain confidence in their ability to implement specialized instructional strategies, manage challenging behaviors, and facilitate student learning despite obstacles. This sustained sense of efficacy is particularly critical for students with disabilities, who often require extended learning time, multiple instructional approaches, and consistent application of evidence-based practices.

The strength of the resilience–efficacy relationship in the Saudi sample may reflect professional norms emphasizing perseverance and role commitment embedded within institutional practice frameworks. Balay-odao et al. (2021) found that Saudi healthcare workers exhibited remarkable resilience during crisis situations, suggesting the presence of systemic and organizational supports that reinforce professional persistence. For special education teachers, this emphasis on sustained professional engagement may manifest as maintained efficacy even under challenging conditions, ultimately benefiting students who rely on consistent and committed instruction.

The non-significant direct relationship between professional resilience and job satisfaction (*β* = 0.089, *p* = 0.126) presents an intriguing paradox that challenges assumptions about teacher well-being. This finding diverges from some Western studies, which typically report positive resilience–satisfaction relationships, but aligns with cross-context research by Huang et al. (2024) and Xu et al. (2025), who identified variation in the institutional and organizational determinants of satisfaction. The absence of a significant relationship suggests that Saudi special education teachers may derive job satisfaction from sources beyond individual resilience. In addition, job satisfaction in reforming systems may depend more strongly on organizational conditions (e.g., leadership support, workload fairness, and professional recognition) than on personal adaptive capacity alone.

This interpretation aligns with Saudi-focused work emphasizing that the effectiveness of inclusive education reforms depends on how support structures are operationalized at the school and system levels (Alharbi and Iqtadar, 2025; Alshahrani and Abu-Alghayth, 2025). It also complements broader evidence linking national reform agendas to well-being outcomes, suggesting that policy implementation and institutional environments can shape occupational well-being (Burger and Arampatzi, 2025).

The partial mediation effects identified in this study illuminate the complex pathways through which contextual factors influence teacher functioning. Notably, compassion fatigue mediated 45.3% of the relationship between work demands and resilience, highlighting compassion fatigue as a key mechanism through which workload pressure erodes adaptive capacity. Similarly, emotional regulation mediated 37.6% of the relationship between personal resources and resilience, indicating that resources are most protective when they translate into improved emotion management. The full mediation observed in the sequential pathways to professional efficacy indicates that work demands and personal resources influence teaching effectiveness entirely through psychological mediators. Practically, this suggests that improving instructional performance under inclusion reforms requires simultaneous attention to (a) reducing chronic stressors and (b) strengthening psychological skills that convert resources into resilience.

These results should be interpreted within the unique educational and institutional reform context of Saudi Arabia. The rapid implementation of inclusive education reforms under Vision 2030 has created both opportunities and challenges for special education teachers (Albash, 2024; Alsolami, 2024). Recent analyses of Saudi inclusive education policy highlight that reforms can expand teachers’ responsibilities and emotional labor, reinforcing the need for institutionally supported well-being frameworks (Alrudayni, 2025; Alharbi and Iqtadar, 2025). However, the high levels of compassion fatigue and work demands observed in this study suggest that contextual and organizational supports, rather than informal cultural coping alone, are essential for protecting teacher well-being. Moreover, the finding that personal resources only partially influence outcomes through emotional regulation highlights the need for targeted professional development tailored to the specific demands of special education practice.

The implications for supporting students with disabilities are clear: promoting teachers’ psychological well-being directly enhances the quality of educational services. When teachers maintain emotional regulation and professional resilience, they can provide the patient, individualized, and emotionally responsive support that students with disabilities require. This includes sustaining high expectations despite slow progress, implementing evidence-based interventions with fidelity, and fostering supportive relationships crucial for student development (Yao and Wang, 2024). Accordingly, inclusive education success under Vision 2030 should incorporate teacher well-being indicators and structured supports as core implementation components rather than optional enhancements (Alrudayni, 2025; Alharbi and Iqtadar, 2025).

It is important to emphasize that the relationships identified in this study should be interpreted as associative rather than causal. Although the proposed structural model is theoretically grounded and supported by robust statistical evidence, the cross-sectional design does not permit conclusions regarding temporal sequencing or causal directionality among work demands, emotional regulation, compassion fatigue, and professional resilience. Accordingly, the observed mediation pathways reflect systematic associations consistent with established psychological theories, rather than definitive causal mechanisms.

## 7 Limitations and future directions

Several limitations should be acknowledged. First, the cross-sectional design does not allow for causal conclusions; longitudinal research is necessary to determine temporal relationships and investigate the evolution of these psychological processes. Longitudinal designs would be particularly useful for capturing changes associated with ongoing inclusive education reforms. Second, the reliance on self-report measures introduces potential common method bias, although the use of validated instruments, satisfactory discriminant validity, and procedural remedies such as anonymity assurance and scale separation partially mitigate this concern. Third, while the sample achieved geographic diversity across Saudi regions, voluntary participation and online data collection may have introduced selection bias, potentially overrepresenting teachers with greater digital literacy or motivation to participate. Fourth, the study focused on individual-level factors without directly measuring organizational and institutional variables that likely influence teacher well-being, such as school leadership practices, workload allocation, and institutional support mechanisms.

Future research should employ longitudinal designs to examine the dynamic interplay between these constructs across academic years. Mixed-methods approaches, incorporating qualitative interviews, could offer more details about institutional and contextual influences on emotional regulation and resilience. Intervention studies testing targeted emotional regulation and resilience training programs would provide valuable evidence for practice. Furthermore, multi-level modeling that integrates school- and district-level variables may augment comprehension of system-level impacts on teacher well-being.

Comparative multi-context studies between Saudi Arabia and other educational systems would elucidate the distinction between universal and context-specific processes. Examining potential moderators, including years of experience, disability specialization, or gender, may reveal subgroups that necessitate targeted support. Finally, examining student outcomes in relation to teacher psychological variables would clarify the broader educational impact of promoting teacher well-being.

## 8 Conclusion

This study examined the psychological factors influencing special education teachers’ capacity to care for students with disabilities in Saudi Arabia. The findings demonstrate that teacher well-being—particularly the ability to manage compassion fatigue and maintain professional resilience through emotional regulation—substantially affects their professional efficacy in serving students with complex needs.

The strong relationships identified between work demands, compassion fatigue, and reduced resilience highlight the risks that systemic pressures pose to the quality of special education services. Conversely, the positive associations among personal resources, emotional regulation, and professional efficacy suggest potential pathways for enhancing the care provided to students with disabilities. These findings underscore the interdependence between supporting students with disabilities and supporting the educators who serve them.

As Saudi Arabia advances its inclusive education agenda under Vision 2030, these results highlight the importance of comprehensive support systems for special education teachers. Interventions aimed at enhancing emotional regulation, managing workload, and preventing compassion fatigue could strengthen teachers’ ability to provide the patient, individualized, and emotionally responsive care that students with disabilities need.

The implications extend beyond individual teacher well-being to the overall quality of special education services. When teachers are psychologically supported and professionally resilient, they are better equipped to fulfill their critical role in helping students with disabilities reach their full potential. This includes maintaining high expectations, implementing evidence-based interventions with fidelity, fostering supportive relationships, and advocating for students’ needs.

Future efforts to improve outcomes for students with disabilities must recognize that teacher psychological well-being is a foundational element of quality special education. By addressing factors that drain teachers’ emotional resources and developing systems that foster resilience and efficacy, educational institutions can ensure that students with disabilities receive not only adequate services but also the compassionate, skilled, and sustained support they need to thrive. Ultimately, this research underscores that caring for students with disabilities is inseparable from caring for those who teach them, making teacher well-being a critical priority in special education policy and practice.

This study examined the psychological factors influencing special education teachers’ capacity to care for students with disabilities in Saudi Arabia. The findings demonstrate that teacher well-being—particularly the ability to manage compassion fatigue and maintain professional resilience through emotional regulation—substantially affects their professional efficacy and instructional effectiveness in serving students with complex needs.

The strong relationships identified between work demands, compassion fatigue, and reduced resilience highlight the risks that systemic pressures pose to the quality of special education services. Conversely, the positive associations among personal resources, emotional regulation, and professional efficacy suggest modifiable pathways for enhancing the care provided to students with disabilities. These findings underscore the interdependence between supporting students with disabilities and supporting the educators who serve them.

As Saudi Arabia advances its inclusive education agenda under Vision 2030, these results highlight the importance of institutionalized and sustainable support systems for special education teachers. Interventions aimed at enhancing emotional regulation, managing workload, and preventing compassion fatigue could strengthen teachers’ ability to provide the patient, individualized, and emotionally responsive care that students with disabilities need within inclusive school environments.

The implications extend beyond individual teacher well-being to the overall quality of special education services. When teachers are psychologically supported and professionally resilient, they are better equipped to fulfill their critical role in helping students with disabilities reach their full potential. This includes maintaining high expectations, implementing evidence-based interventions with fidelity, fostering supportive relationships, and advocating for students’ needs across diverse educational settings.

Future efforts to improve outcomes for students with disabilities must recognize that teacher psychological well-being is a foundational element of quality special education. By addressing factors that drain teachers’ emotional resources and developing systems that foster resilience and efficacy, educational institutions can ensure that students with disabilities receive not only adequate services but also the compassionate, skilled, and sustained support they need to thrive. Ultimately, this research underscores that caring for students with disabilities is inseparable from caring for those who teach them, positioning teacher well-being as a strategic priority in inclusive education policy and practice.

## Data Availability

The raw data supporting the conclusions of this article will be made available by the authors, without undue reservation.
